# Sporadic pancreatic desmoid-type fibromatosis with curative resection: A case report

**DOI:** 10.1097/MD.0000000000041924

**Published:** 2025-03-21

**Authors:** Jiayao Zhang, Chenming Liu, Fangzheng Zhou, Baochun Lu

**Affiliations:** a Department of Hepatobiliary and Pancreatic Surgery, Shaoxing People’s Hospital, Shaoxing, China; b Zhejiang University School of Medicine, Hangzhou, China; c School of Medicine, Shaoxing University, Shaoxing, China.

**Keywords:** desmoid tumor, desmoid-type fibromatosis, pancreas, treatment

## Abstract

**Rationale:**

Desmoid-type fibromatosis (DF) is an uncommon, locally invasive, non-metastatic soft-tissue neoplasm with variable and unpredictable manifestations. The therapeutic arsenal of DF therapy is consistently expanding; however, there remains no standard treatment modality. Sporadic pancreatic DF is rarely described in current literature, reflecting a significant deficiency in clinical treatment experience, this case aims to share some clinical experiences that can serve as a reference for managing this rare disease.

**Patient concerns:**

A 36-year-old male presented with occasional abdominal discomfort and weight loss over a year. Ultrasound revealed a large mass in the pancreatic tail, which was not observed a year ago.

**Diagnoses:**

The diagnosis of DF was confirmed by immunohistochemistry nuclear staining of β-catenin.

**Interventions:**

Distal pancreatectomy with splenectomy was performed and the patient received no further therapy.

**Outcomes:**

After 13 months of follow-up, no recurrence or distant metastasis was observed.

**Lessons:**

DF is a distinct rare tumor entity, sporadic pancreatic DF is even rarer. It is imperative to select the individualized treatment strategy for each patient to optimize tumor control and enhance quality of life.

## 1. Introduction

Desmoid-type fibromatosis (DF), also known as desmoid tumor (DT), constitutes an extremely rare soft-tissue neoplasm, accounting for only 0.03% of all neoplasms. It has a low incidence rate, affecting 2 to 5 per million people, and is more common in young female.^[1,2]^ The exact etiology of DTs is unclear, but it is closely linked to familial adenomatous polyposis (FAP). DTs occur in about 5% to 10% of FAP cases,^[1,3]^ making FAP-associated and sporadic DTs the 2 main types. DTs originate from any fibrous connective tissues throughout the body and are mainly categorized into extra-abdominal, abdominal wall, and intra-abdominal types.^[4]^ Intra-abdominal DTs are rare and typically linked to FAP (Gardner syndrome). Only 5% of sporadic cases are intra-abdominal,^[1]^ with mesenteric or retroperitoneal origins, and pancreatic DTs are particularly uncommon.

Here we presented a case of isolated and sporadic pancreatic DF in a male patient, who underwent curative resection alone. No recurrence or metastasis was observed 13 months following surgery.

## 2. Case report

A 36-year-old male has intermittently experienced mild to moderate abdominal discomfort, along with a slight weight loss over the past year, with no prior history of trauma or surgery, or family history. An ultrasound detected a large solid mass at the pancreas’ tail, which was not observed a year ago. Physical and laboratory tests revealed no significant issues, and gastrointestinal endoscopy was normal.

**Figure 1.**
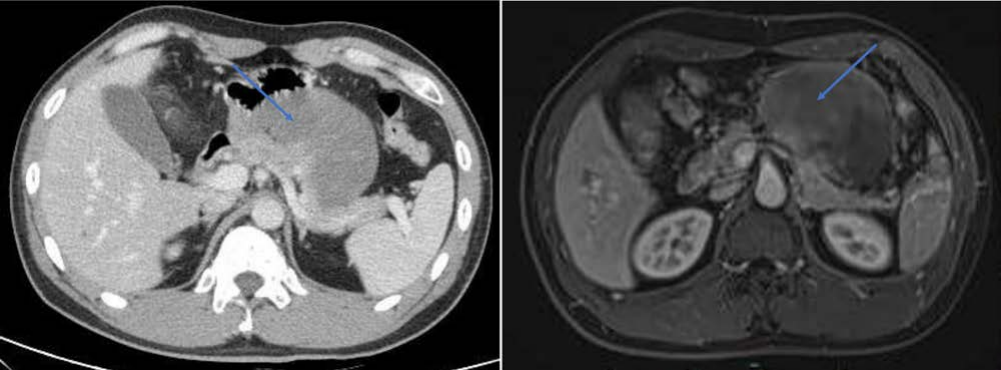
Imaging investigations: a heterogeneous and solid mass in the pancreatic tail which is well-delimited with fat signal in the internals.

**Figure 2.**
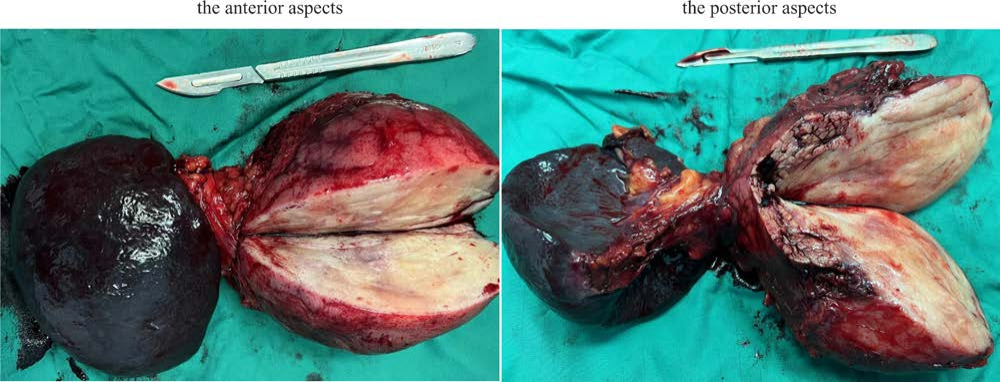
Surgical specimen: the mass measures approximately 10 × 8 × 9 cm and the cut surface is heterogeneous yellow to white.

**Figure 3.**
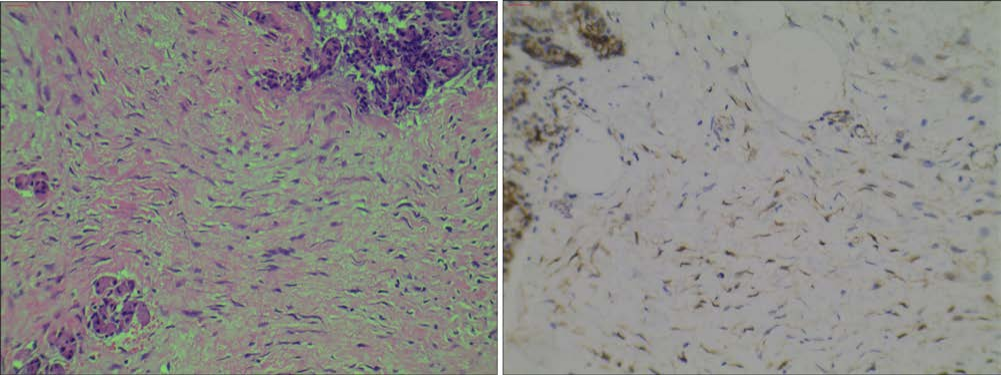
Histology and immunohistochemical: the tumor cells were spindle-shaped and interspersed with collagen matrix with diffuse nuclear expression of β-catenin.

Abdominal enhanced computed tomography scan revealed a 97 × 72 mm heterogeneous mass at the pancreas tail, well-defined with internal fat signal, and no vascular invasion or metastasis. In conjunction with the magnetic resonance imaging scan confirmed suspected pancreatic liposarcoma diagnosis (Fig. 1). Extensive adhesion to adjacent organs was found through surgical exploration and intraoperative frozen section indicated spindle cell tumor. An en bloc resection of the distal pancreas with the adherent spleen, with partial resection of the adhesive stomach and duodenum wall was performed. The surgical procedure was smooth. All surgical resection margins were negative. Gross examination of the surgical specimen revealed a yellowish-white nodular mass, approximately 10 × 8 × 9 cm, closely related to the pancreatic parenchyma (Fig. 2). Microscopic specimen showed uniform spindle cells and dense collagen matrix without marked necrosis and atypia, aligning with the characteristics of DF.^[4]^ Pathological diagnosis confirmed primary pancreatic DF, as tumor cells were positive for β-catenin in the nuclei (Fig. 3).^[1,3,4]^ The patient was discharged on day 13 without adjuvant therapy and no significant adverse events were recorded. During 13 months of follow-up, no local recurrence or distant metastasis was identifiable.

## 3. Discussion

To date, no consensus exists on DTs treatment, with options like active surveillance (AS), surgery, radiotherapy, and systemic therapy. In recent years, a paradigm shift in the management of DTs had certainly taken place. AS had been broadly adopted in many countries as the initial approach.^[1,3,5–9]^ A prospective multi-center study evaluated the behavior of 108 patients with primary sporadic DTs initially treated with AS, which found that spontaneous regression occurred in half of the patients.^[5]^ A mounting body of clinical studies provided evidence to support AS as a first-line approach for DTs and recommend avoiding unnecessary treatment or surgery. However, in our case, we decided to proceed with surgical resection and attempted to achieve a negative margins without adjuvant treatment, balancing surgical feasibility and organ function preservation. Our approaches are based on 2 main considerations.

### 3.1. Surgery: the decision dilemma

1. For asymptomatic DTs patients, AS is recommended as initial treatment, while progressive symptoms and persistent interval growth are indications for intervention.^[6]^ Our patient occasionally experienced mild to moderate abdominal discomfort, accompanied by slight weight loss, as the mass enlarged to 97 × 72 mm over the course of a year.
2. Given that the peak age range of 30 to 40 years, this group particularly requires comprehensive programs including physical, psychological, and social support.^[3]^ Quality of life is also seen as one of the priority factors in the managing of DTs.^[6]^ The 36-year-old patient, who was previously in good health, experienced significant tension and anxiety following the discovery of a mass. This adversely impacted daily life and sleep quality, prompting a strong desire for surgery.
3. The prognosis of DTs is highly unpredictable. A retrospective study identified 168 patients with primary DTs who were initially managed with AS. Among them, 36% experienced radiological progressive disease, 36% had stable disease, and 27% showed regressed,^[7]^ which was similar to the findings of a recent single-institution study by Bartholomew (28% stabilization, 33% regression, and 39% progression).^[8]^ In addition, a study of prognostic factors affecting DTs progression-free survival indicated that youth (<37 years; HR: 1.97; 95% CI: 1.36 ~ 2.84; *P* = .01) and tumor size (>7 cm; HR: 1.64; 95% CI: 1.13 ~ 2.36; *P* = .008) were associated with poor outcomes.^[9]^ Our patient presented with an unidentified pathological condition prior to surgery. Intraoperative frozen section analysis indicated a spindle cell tumor, prompting a radical resection. The diagnosis of DF was confirmed by immunohistochemistry showing nuclear β-catenin. We maintain that a radical resection remains essential for the patient, as he exhibits two adverse prognostic factors that signify a heightened risk of disease progression, and preoperative imaging evaluation has confirmed the resectability of the tumor. A consensus holds that employing 14G or 16G needles in core biopsy can readily establish the diagnosis of DF, and it is not recommended to use incisional or excisional biopsy as the initial diagnostic approach.^[3]^ Preoperative definitive diagnosis is beneficial for the clinical management of DF, but a biopsy was not performed in our case, which constitutes a limitation.

### 3.2. Operational methodology and subsequent adjuvant therapy regimen are key

1. The link between resection margins and recurrence rates is debated. A meta-analysis reported resected with microscopically positive margins had a twofold increase in recurrence rate (R0 *v* R1; risk ratio: 1.78, 95% CI: 1.40 ~ 2.26).^[10]^ Nevertheless, the progression-free survival curves of patients with DTs of a prospective study were not significantly different between R0 and R1 resection (*P* = .87) but significantly different between R2 and R0/R1 resection (*P*<0.001).^[9]^ A updated European Consensus Initiative argued that even if R1 or R2 resection status was performed, a re-resection should not be routinely considered and AS was preferential approach to minimize overtreatment and unnecessary morbidity.^[3]^ A study demonstrated that adjuvant radiation therapy (RT) after R1 resection surgery reduces recurrence rates in patients with recurrent tumors, it does not have the same effect following R0 resection.^[10]^ Besides, RT is inappropriate for intra-abdominal DTs due to potential radiation-induced toxicity (e.g., RT-related fibrosis and secondary malignancies).^[11]^ Consequently, we opted for AS as the primary treatment without RT for subsequent follow-up. The AS for our patient consisted of enhanced computed tomography scans at 1, 2, and 3 months after surgery, then every 3 months until year 3, and twice yearly thereafter. Meticulous observation is essential, accompanied by timely intervention should disease progression be detected.
2. In recent years, systemic therapy has played a significant role in the management of DTs, including antiestrogens, nonsteroidal anti-inflammatory drugs, cytotoxic chemotherapy, tyrosine kinase inhibitors, and γ-secretase inhibitors. Anti-inflammatory agents and hormonal therapy are debated because evidence is almost based on small case series or reports and prospective studies are limited. Cytotoxic agents and tyrosine kinase inhibitors (e.g., sorafenib) are widely recognized as the principal medical treatments, γ-secretase inhibitors (e.g., nirogacestat) are even granted approval by the US Food and Drug Administration for the management of DTs after the DeFi trial, potentially becoming the preferred first-line treatment when active medical intervention is needed.^[4,12]^ There is an ineligible issue that these drugs have some on-treatment side effects (e.g., rash, permanent hypertension, diarrhea, ovarian dysfunction).^[13,14]^ Their long-term safety has never been assessed, and our patient is young with a normal life expectancy. Thus, we do not recommend that systemic therapy is eagerly needed for our patient unless surveillance fails or recurrence after surgery occurs. Quality of life, both immediate and long-term, should be the main dimensions to weigh when the strategies (surgery or adjuvant therapy) are decided.

## 4. Conclusions

In conclusion, surgical resection is the preferred treatment for symptomatic patients with resectable sporadic pancreatic DF that affect their quality of life and pose a risk of progression or poor prognosis. sporadic pancreatic DF is a rare soft-tissue neoplasm with invasive growth that is locally quite destructive, posing significant clinical challenges. In recent years, there is a paradigm shift in the management of DTs, AS is generally considered the first-line approach. However, we hold that optimal treatment is not standardized and needs to be individualized on a multi-modal basis, necessitating ongoing research and international collaboration. It is necessary to carefully select the management strategies for each patient with DF to improve quality of life.

## Acknowledgments

We would like to thank all of our colleagues in the medical and nursing team of the hepatobiliary and pancreatic surgery.

## Author contributions

**Conceptualization:** Jiayao Zhang, Chenming Liu, Fangzheng Zhou.

**Writing – original draft:** Jiayao Zhang.

**Writing – review & editing:** Baochun Lu.
